# Thoracic hyperextension injury with complete “bony disruption” of the thoracic cage: Case report of a potentially life-threatening injury

**DOI:** 10.1186/1749-7922-7-14

**Published:** 2012-05-15

**Authors:** James Bailey, Todd VanderHeiden, Clay Cothren Burlew, Sarah Pinski-Sibbel, Janeen Jordan, Ernest E Moore, Philip F Stahel

**Affiliations:** 1 Department of Orthopaedics, Naval Medical Center, San Diego, CA, 92134, USA; 2 Department of Orthopaedics, Denver Health Medical Center, University of Colorado School of Medicine, 777 Bannock Street, Denver, CO, 80204, USA; 3 Department of Surgery, Denver Health Medical Center, University of Colorado School of Medicine, 777 Bannock Street, Denver, CO, 80204, USA

## Abstract

**Background:**

Severe chest wall injuries are potentially life-threatening injuries which require a standardized multidisciplinary management strategy for prevention of posttraumatic complications and adverse outcome.

**Case presentation:**

We report the successful management of a 55-year old man who sustained a complete “bony disruption” of the thoracic cage secondary to an “all-terrain vehicle” roll-over accident. The injury pattern consisted of a bilateral “flail chest” with serial segmental rib fractures, bilateral hemo-pneumothoraces and pulmonary contusions, bilateral midshaft clavicle fractures, a displaced transverse sternum fracture with significant diastasis, and an unstable T9 hyperextension injury. After initial life-saving procedures, the chest wall injuries were sequentially stabilized by surgical fixation of bilateral clavicle fractures, locked plating of the displaced sternal fracture, and a two-level anterior spine fixation of the T9 hyperextension injury. The patient had an excellent radiological and physiological outcome at 6 months post injury.

**Conclusion:**

Severe chest wall trauma with a complete “bony disruption” of the thoracic cage represents a rare, but detrimental injury pattern. Multidisciplinary management with a staged timing for addressing each of the critical injuries, represents the ideal approach for an excellent long-term outcome.

## Background

Blunt chest injuries represent a major cause of preventable mortality after trauma [1-3]. Serial rib fractures or a flail chest, in conjunction with a fractured sternum and unstable fractures of the thoracic spine, can lead to a complete “bony disruption” of the thoracic cage [4]. This entails a discontinuation of the chest wall integrity and muscular support, which is, most importantly, required for breathing and sufficient ventilation. While such critical injuries are rare, they pose a potential life-threatening risk related to underlying pulmonary contusions, impaired ventilatory mechanics, and the risk of developing posttraumatic complications and adverse pathophysiological sequelae [2,4]. These include the development of ventilator-associated pneumonia, acute respiratory distress syndrome, and subsequent multiple organ failure and death [5]. Some authors advocate for early rib fixation in patients with a flail chest, in order to restore the physiological ventilation impaired by the “paradoxical breathing” associated with segmental rib fractures [6,7]. In addition, unstable thoracic spine fractures are associated with a high risk for neurologic injury, particularly in younger victims and high-energy trauma mechanisms [8,9]. Early spine fixation for patients with unstable thoracic spine fractures results in a decreased incidence of respiratory complications [10-13].

In the present case report, we describe a successful management strategy for a complete “bony disruption” of the thoracic cage, in conjunction with a displaced transverse sternum fracture and an unstable hyperextension injury of the thoracic spine.

## Case report

**Figure 1:**
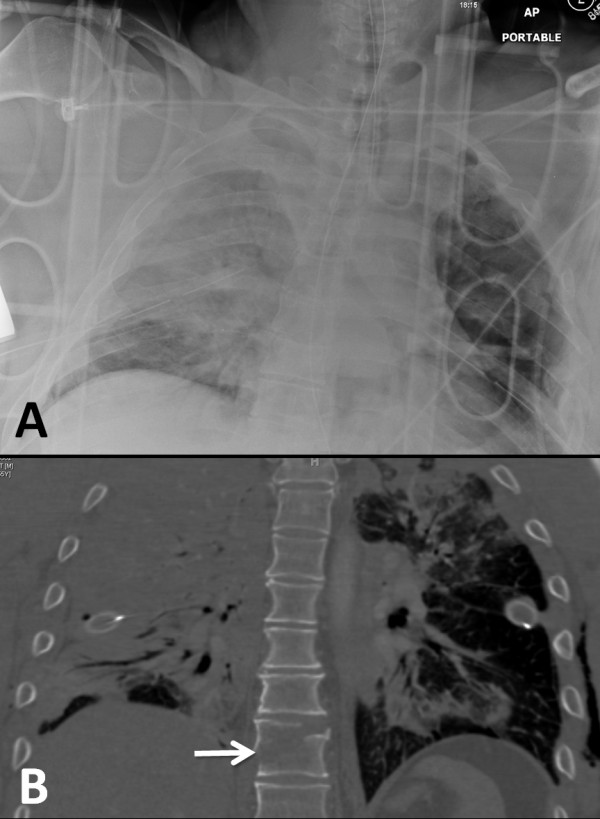
**Initial chest radiograph (A) and coronal CT scan reconstruction (B) on arrival in the emergency department.** Despite placement of bilateral chest drains, there is a persistent, extensive hemothorax on the right side, and signs of bilateral lung contusions. The arrow in **panel B** points out the T9 hyperextension injury in the coronal plane.

**Figure 2:**
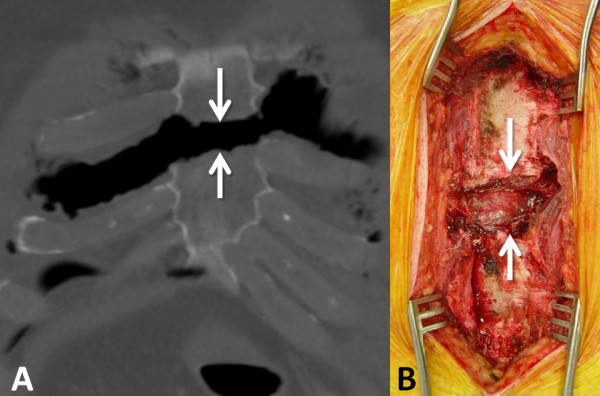
**Displaced transverse sternal fracture in coronal CT scan (A) and operative site (B) after exposure for the sternal fracture fixation procedure.** The arrows point out the impressive fracture diastasis of about 3 cm, with the retrosternal pericardium exposed in **panel B**.

**Figure 3:**
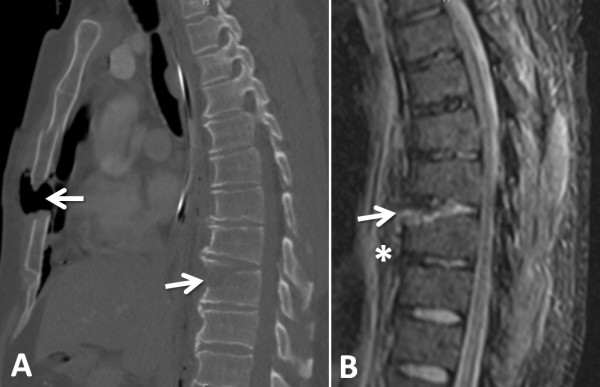
**Sagittal CT scan (A) and STIR sequence in MRI (B) of the T9 hyperextension injury (arrows).** The asterisk in **panel B** alludes to the extensive prevertebral hematoma.

A 55-year-old man was involved in a helmeted “all-terrain vehicle” (ATV) roll-over accident. He had a loss of consciousness and a prolonged extrication, since his body was pinned to the ground by the ATV. The patient was found to be comatose and in respiratory arrest, with a Glasgow Coma Scale (GCS) score of 3. He was endotracheally intubated at the accident scene and transferred to a local hospital in the Rocky Mountain region. On arrival, he was found to be hypotensive and tachycardic, with a blood pressure of 82/54 mmHg, a heart rate of 136 bpm, and SO_2_ of 96% (on 100% FiO_2_). The initial laboratory work-up showed a hemoglobin level of 8.2 g/dL, INR of 1.2, PTT of 30.1 s, pO_2_ of 35 mmHg, base excess of 1.1 mEq/L, and lactate of 1.6 mmol/L. Chest radiograph revealed bilateral pneumo-hemothoraces, which were managed by placement of bilateral chest tubes, prior to airlift evacuation to our level 1 trauma center. After initial assessment and management by ATLS® protocol in our emergency department [14], the patient was transferred to the surgical intensive care unit (SICU) for ongoing resuscitation and ventilatory management. After radiologic workup by conventional films and “total body” computed tomography (CT) scan, the patient was diagnosed with the following injury pattern (Figure 1,2,3):

Severe chest trauma with bilateral “flail chest” with serial segmental rib fractures (C1-8 on right side, C1-10 on left side), bilateral pulmonary contusions, and bilateral hemo-pneumothoraces, a displaced transverse sternum fracture with 3 cm diastasis, bilateral midshaft clavicle fractures, and an unstable T9 hyperextension injury.

The unstable T9 fracture was associated with a chronic hyperostotic ankylosing condition (“diffuse idiopathic skeletal hyperostosis”; DISH) of the thoracic spine, as revealed in the sagittal CT scan reconstruction (Figure 3A). An MRI of the T-spine was obtained to further assess for an associated disc or ligamentous injury, and to rule out the presence of an epidural hematoma, any of which may alter the surgical plan and modality of spinal fixation or fusion.

**Figure 4:**
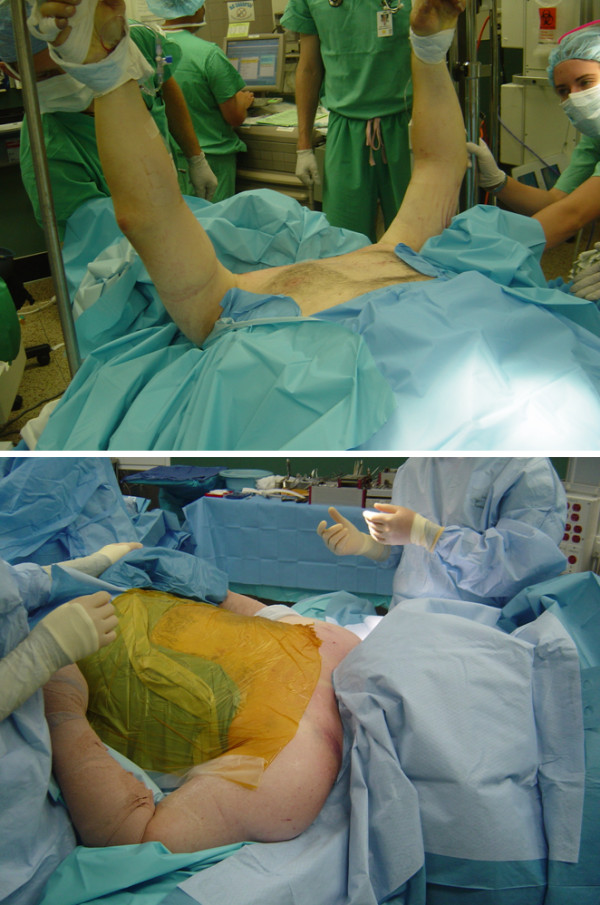
Technique of patient positioning and draping for surgical fixation of the bilateral clavicle fractures and the displaced sternal fracture.

After resuscitation in the SICU, and adequate thoracic pain control by epidural anesthesia, the patient was taken to the OR on day 4 for fracture fixation. A decision was made for surgical fixation of bilateral clavicle fractures, the sternal fracture, and the T9 spine fracture, in order to achieve adjunctive stability of the thoracic cage and to allow early functional rehabilitation without restrictions. The patient was placed on a radiolucent flat-top operating table in supine position. The technique of positioning, preparation and draping, aimed at addressing both clavicle fractures and the sternum fracture in one session, are depicted in Figure 4.

**Figure 5:**
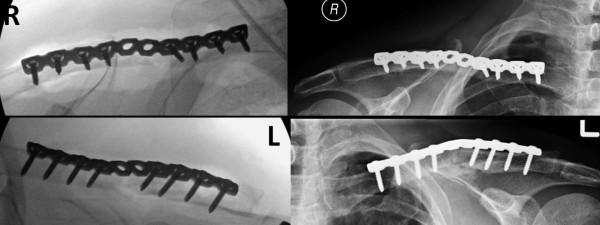
Intraoperative fluoroscopy films of bilateral clavicle fracture fixation in bridging technique (left panels), and follow-up radiographs at 6 months, demonstrating the bilateral healed fractures (right panels).

**Figure 6:**
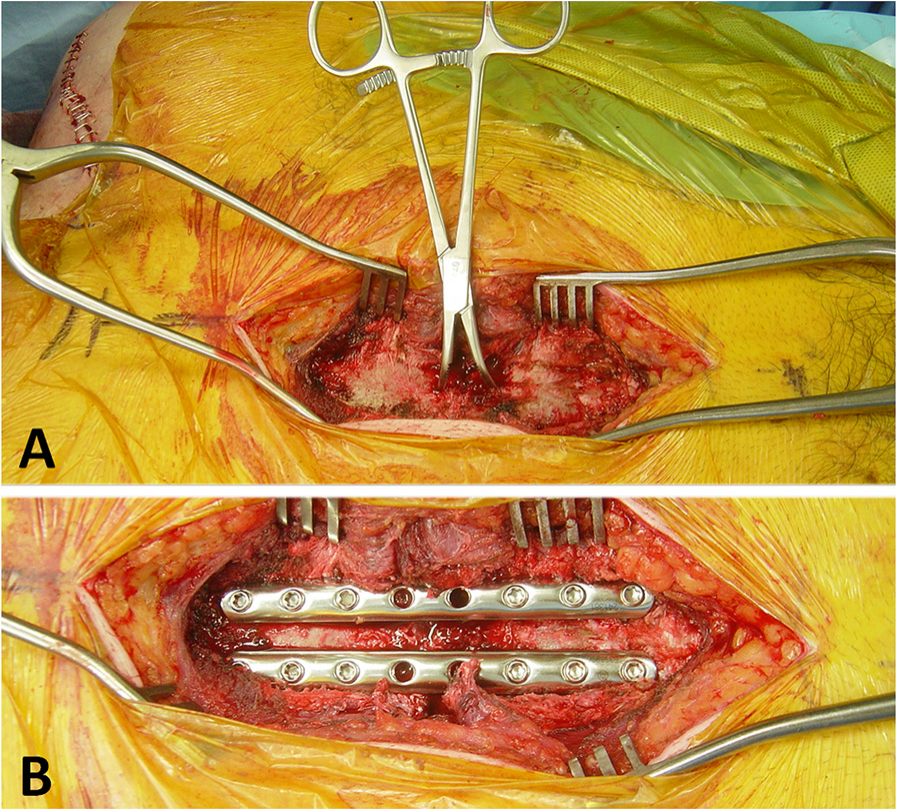
**Intraoperative view of the technique for fracture reduction (A) and locked plating (B) of the displaced transverse sternum fracture.** See text for details.

The bilateral comminuted clavicle fractures were fixed through an S-shaped subclavicular approach, using two 10-hole 3.5 mm reconstruction plates (Synthes, West Chester, PA), which were applied in bridging technique (Figure 5). This was followed by a median approach to the transverse sternal fracture. The sternum had a diastasis of about 3 cm through which the mediastinal fat pad and pericardium was evident (Figure 2B). The video clip in the Additional file 1 shows the beating heart behind the sternal fracture. A 2.5 mm unicortical hole was drilled on each side of the fracture, to allow placement of a pointed reduction tenaculum for anatomic reduction of the sternal fracture (Figure 6A). The fracture was then fixed with two 8-hole 3.5 mm third-tubular locking plates (Synthes), using unicortical locking head screws. This technique was used to avoid screw penetration across the far cortex, with the risk of a delayed arrosion of the pericardium (Figure 6B).

**Figure 7:**
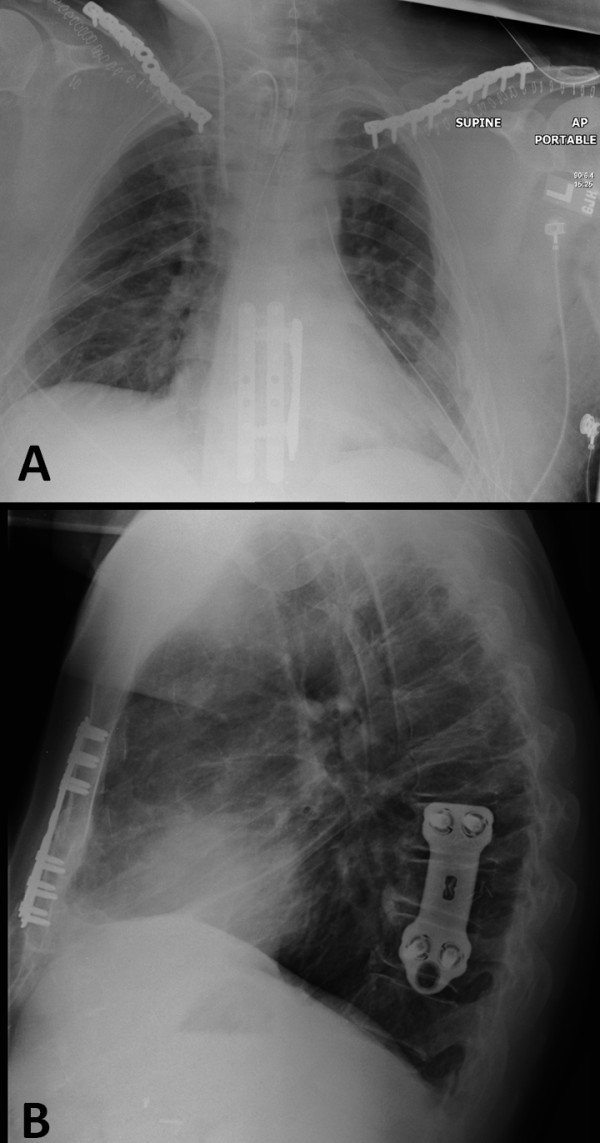
Radiographic documentation demonstrating the sternal fracture and T9 spine fixation in antero-posterior chest X-ray (A), and in the lateral plane at 6 months follow-up (B).

After wound closure, the patient was carefully log-rolled into a right lateral decubitus position on a pre-positioned beanbag, for operative fixation of the unstable T9 vertebral fracture. Two-level spinal fixation from T8-T10 was performed using a titanium locking plate system (THOR™, Stryker, Allendale, NJ), through a less-invasive postero-lateral approach, as previously described [15]. A tracheostomy was performed in the same session, due to the requirement of prolonged ventilation in the SICU. The postoperative chest radiographs demonstrates the plate fixation of bilateral clavicles, sternum, and thoracic spine (Figure 7A). The patient tolerated the surgical procedures well and remained hemodynamically stable throughout the case. He was weaned from mechanical ventilation, and the chest tubes were appropriately removed. The patient was transferred to an acute rehabilitative facility on postoperative day 16.

The patient was readmitted three weeks later, 6 weeks post injury, for acute fever, chills, and night sweats, in conjunction with increased oxygen requirement. A right-side chest drain was placed which showed purulent drainage, and the patient was diagnosed with a pleural empyema, likely related to a retained hemothorax. He underwent a video-assisted thoracoscopic pleural decortication. Two 32 French pleural chest drains were placed intraoperatively. The patient recovered well from the procedure, and he was treated with adjunctive antibiotics. The chest drains were removed prior to discharge within a week of surgery.The patient was discharged home in good condition.

All surgical wounds healed uneventfully, and there were no further complications. Within three months after the accident, the patient had returned to exercising without restrictions and was able to hike a mountain with altitude above 14,000 ft, with minimal subjective shortness of breath. At 6 months follow-up, X-rays revealed a fully healed sternal fracture, T9 vertebral fracture (Figure 7), and bilateral clavicle fractures (Fig.5). The patient had a full range of motion in bilateral shoulders and in the T- and L-spine, and a normal neurovascular status in all four extremities. He was released to full activity without restrictions, and scheduled to follow-up as needed.

## Discussion

The structural support of the thoracic cage is provided by the sternum in conjunction with the rib cage and the thoracic spine [16,17]. The adjunctive anterior support for the thoracic spine by the sternum has been accurately described as “the 4^th^ spinal column” by Berg in 1993 [18], in modification of Denis’ classic “three column model” of spinal stability [19]. The thoracic cage stability is further bolstered by clavicular strut attachments to the sternum and a complex interplay between the clavicles and the scapulae as they attach to the posterior thorax [20]. High-energy trauma mechanisms to the chest and thoracic spine can result in critical injuries, including pulmonary and cardiac contusions, aortic injuries, and acute spinal cord injuries [21]. Unstable thoracic spine injuries typically result from flexion/distraction or hyperextension injuries in association with a sternal fracture, representing the classic “4-column thoracic spine fracture” [18,22-24]. These combined fractures often occur in high-energy, multi-system trauma, and can be easily overlooked on initial evaluation [25,26].

The present case reports describes the successful management of a severe chest trauma in a 55 year-old patient who sustained a complete “bony disruption” of the thoracic cage, consisting of bilateral segmental serial rib fractures (“flail chest”), bilateral comminuted clavicle fractures, an unstable T9 hyperextension injury, and a displaced transverse sternal fracture. The combination of early fracture fixation, in conjunction with modern ventilatory and pain management strategies in the SICU, allowed for an excellent long-term outcome.

The “ideal” timing and modality of managing a complete “bony disruption” of the chest wall remains controversial. The surgeon in charge should consider (1) the acute life-threatening aspects of the injury, related to airway compromise and ventilatory management; (2) the timing of definitive surgical fracture fixation in relation to the acute hyperinflammatory response, taking into account the risk of sustaining a “2^nd^ hit” injury related to a prolonged surgical intervention [27]; (3) the anatomic and functional relation of the rib cage with the shoulder girdle and the thoracic spinal column, which provide the underlying rationale for the specific modality of surgical fracture fixation [8,17,20].

The primary objective of the initial management of multiply injured patients is *survival*. The acute management by “damage control” procedures will limit the extent of the operative and interventional burden, and allow early patient transfer to the SICU, for full resuscitation [14]. The pathophysiological disturbances of the immune and clotting systems render multiply injured patients vulnerable to “2^nd^ hit” insults related to inadequate timing and modality of surgical procedures [27]. The ideal timing for definitive fracture fixation lies in a limited physiological “time-window of opportunity”, somewhere around day 4 to 10 after trauma [11,14].

From a biomechanical perspective, the surgeon must take into consideration the “four-column model” of thoracic stability [18,28,29] provided by the rib-cage and the thoracic spine, in conjunction with the shoulder balance provided by clavicular strut integrity [16,17,22,30,31]. The present case report outlines the biomechanical importance of the integrity of the “upper transthoracic cage” [4], based on the functional interaction between the shoulder girdle, the rib cage, and the thoracic spine.

Notably, sternal fractures are frequently missed in the trauma bay, since dedicated sternum radiographs are not part of the standard trauma work-up. Based on the important biomechanical aspects related to thoracic cage integrity outlined above, missed sternal fractures in conjunction with upper thoracic spine injuries can have significant adverse effects, including respiratory distress and pulmonary complications, neurological compromise to the spinal cord, chronic pain, malunion, and progressive kyphotic deformity [4,8,23,26,32,33].

Multiple technical modalities for sternal fixation have been described in the literature [34], including wiring, conventional plating, threaded pin fixation, flexible intramedullary nailing [33]. Locked plating of sternal fractures and sternal nonunions has been previously described, by the use of designated sternal locking plates, anterior cervical locking plates, and mandibular locking plates [35-37]. However, the technique of using two parallel stainless-steel tubular locking plates applied in the present case has not been previously described in the literature, to our knowledge [34]. We believe that this represents a feasible, safe, and cost-effective strategy which results in excellent outcome, as reflected by this case report.

In conclusion, we present a safe and successful strategy for managing a highly unstable and potentially life-threatening disruption of the chest wall, associated with a “four-column” hyperextension injury of the thoracic spine in conjunction with a displaced transverse sternal fracture. A standardized multidisciplinary resuscitation protocol [14], in conjunction with modern ventilatory management strategies in the SICU [2,38] and a proactive surgical fixation of the “bony disruption” of the chest wall during the physiological time-window of opportunity [11,14], likely contributed to the excellent long-term outcome of this critically injured patient.

## Competing interests

The authors declare that they have no competing interests.

## Authors’ contributions

PFS, TVH, and CCB designed the case report. PFS, CCB, SSP, and JJ performed the surgical procedures in this patient. JB drafted the first version of the manuscript. PFS and EEM critically revised this paper. All authors contributed and approved the final version of the manuscript.

## Disclosures

PFS has obtained occasional speaker’s honoraria from Stryker Spine (Allendale, NJ) within the past 5 years. The authors declare no other competing interests related to this manuscript. The views expressed in this article are those of the authors and do not reflect the official policy or position of the Department of the Navy, Department of Defense, or the United States Government.

## Supplementary Material

Additional file 1 Intraoperative video clip of beating heart exposed by the displaced transverse sternum fracture. Click here for file
